# Distinct clinical characteristics of bocavirus and *Mycoplasma pneumoniae* infection in children plastic bronchitis

**DOI:** 10.1002/iid3.1373

**Published:** 2024-08-16

**Authors:** Mengqi Liu, Diwei Wei, Tongqiang Zhang, Yongsheng Xu, Wei Guo

**Affiliations:** ^1^ Department of Respiratory, Tianjin Children's Hospital (Children's Hospital, Tianjin University), Tianjin Pediatric Research Institute Tianjin Key Laboratory of Birth Defects for Prevention and Treatment Tianjin China; ^2^ Children's Clinical College of Tianjin Medical University Tianjin China

**Keywords:** flexible bronchoscope, human bocavirus, infection, *Mycoplasma pneumoniae*, plastic bronchitis

## Abstract

**Background:**

This study investigated clinical and laboratory characteristics of human bocavirus type 1 (HBoV1)‐plastic bronchiolitis (PB), *Mycoplasma pneumoniae* (MP)‐associated plastic bronchitis (PB) and MP‐NPB in children, highlighting inflammation, coagulation, and bronchoscopic needs.

**Methods:**

Data on preschool children with PB during HBoV1 or MP infection were collected, comparing MP‐PB to severe *Mycoplasma pneumoniae pneumonia*.

**Result:**

Compared with the MP‐PB group, the HBoV1‐PB group, with younger children, had significantly milder clinical symptoms but higher WBC counts (*p* = .028). The MP‐PB group exhibited notably elevated Fibrinogen (*p* = .045) and d‐dimer levels (*p* < .001). When contrasting the MP‐PB with the MP‐NPB group, children in MP‐PB group still had higher levels of d‐dimer and increased inflammatory indicators such as C‐reactive protein, procalcitonin, lactate dehydrogenase, and interleukin‐6, which were significantly elevated compared with the MP‐NPB group. MP‐PB showed a higher prevalence of plastic bronchial casts in lower lobes (*p* = .016) and a dominance of neutrophils in BALF cytology. Additionally, children in the MP‐PB group tended to undergo a greater number of bronchoscopies.

**Conclusion:**

This study identifies key differences in plastic bronchitis in children due to HBoV1 and MP, highlighting HBoV1's milder inflammation in younger kids and MP's link to severe inflammatory and coagulation responses, guiding clinical diagnosis and treatment.

## Graphical abstract

This study aimed to analyze the clinical features of human bocavirus type 1 and *Mycoplasma pneumoniae* infection in preschool children diagnosed with plastic bronchiolitis. We found that different pathogens may lead to plastic bronchitis in children through completely different pathogenic pathways.

## 1 INTRODUCTION

Plastic bronchitis (PB), which is characterized by the formation of phlegm‐like obstructions along its course in the bronchi, is a rare respiratory disease.^1^ It can be divided into two types according to its pathologic composition; type I is mainly caused by inflammatory diseases and is composed mainly of inflammatory cells with fibrin; type II is associated with congenital heart disease and is composed mainly of mucin and contains few cells.^2^

Children with substantial plugs are at a higher risk of life‐threatening situations such as acute respiratory distress or dyspnea due to inadequate sputum evacuation. Research suggests that lymphatic vessel abnormalities, viral or bacterial infections, hematologic disorders, occupational exposures, malignancies, and surgery‐related complications may contribute to the formation of casts.^3^ Infection is one of the most important factors leading to type I PB. According to the existing reports, the pathogens of PB in children include adenovirus,^4^ mycoplasma,^5^ influenza virus,^6^ Human bocavirus (HBoV)^7^ and *Bordetella parapertussis*.^8^ In some recent studies, academics believe that a history of allergies and asthma are also closely related to the development of casts in children.^9^, ^10^ *Mycoplasma pneumoniae* (MP) is a common pathogen causing lower respiratory tract infections in children. However, some children with MP infections have poor outcomes despite receiving systemic glucocorticoid treatment to suppress the inflammatory response.^11^ During a bronchoscopy examination, cast‐like plugs can be observed. With advancements in pathogen detection technology, we have identified several cases of PB caused by human bocavirus type 1 (HBoV1) in a short period of time. Human Bocavirus belongs to the *Parvoviridae* family, of which type 1 is commonly in the respiratory infection and type 2 is observed in gastrointestinal infections. PB related to HBoV1 showed slight differences in clinical manifestations and findings compared to children with MP‐PB.

In this study, we compared and analyzed clinical features, computed tomography (CT) scans, laboratory data, and bronchoalveolar lavage fluid (BALF) examinations in children with PB caused by HBoV1 and MP. Our aim is to provide insights for early recognition of HBoV1‐PB and MP‐associated plastic bronchitis (MP‐PB), and explore the possible different mechanisms of the two pathogens, offering new perspectives for rapid diagnosis and treatment in clinical practice.

## 2 METHODS

### 2.1 Study population

We retrospectively reviewed pediatric cases diagnosed with plastic bronchitis or severe *Mycoplasma pneumoniae* pneumonia through flexible bronchoscopy at the Respiratory Department of Tianjin Children's Hospital from December 2022 to August 2023.

Inclusion Criteria: (1) Patients who underwent bronchoscopy. (2) Patients with complete clinical data, appropriate laboratory and CT results. (3) Bronchoalveolar lavages specimens were obtained for pathogenic and cytological examination. (4) Patients who tested positive only for HBoV1 or MP. (5) Patients with Type I plastic bronchitis identified by surgical specimens were included in the PB group.

Exclusion criteria: (1) Children with congenital heart disease, asthma, cystic fibrosis or aspergillosis. (2) Patients who had taken any form of glucocorticoids before hospital admission. (3) Mixed infections were not considered.

The criteria for determining whether a patient should undergo flexible bronchoscopy were based on expert consensus in the relevant field in China.^12^

### 2.2 Laboratory blood tests

Blood specimens were collected from patients within 3 h of admission and sent to the Laboratory Department for routine tests. Results of blood routine, liver function and preoperative coagulation‐related tests were collected and recorded.

### 2.3 Flexible bronchoscopy

Patients received lidocaine as a local anesthetic in the tracheal eminence and vocal folds. Casts were localized according to the patient's CT findings. Forceps for foreign bodies were used to remove the cast. Local irrigation with 0.9% saline was performed before and after using foreign body forceps, and the first three times of irrigation as BALF were collected for cytological and pathogenetic examination. BALF pathogen detection included MP‐DNA, MP‐RNA, and target next‐generation sequencing (tNGS).

### 2.4 Bronchoalveolar lavage fluid examination

BALF was subjected to Wright‐Giemsa staining, followed by microscopic sorting and counting of neutrophils, eosinophils, lymphocytes, macrophages, and epithelial cells. The results were presented as percentages.

BALF was collected for targeted tNGS to further identify the pathogen through the second‐generation sequencing method. Report rules: The original data volume was ≥100 K. (1) Strong pathogenic bacterium such as tuberculosis can be reported directly after the pollution is eliminated. If pollution cannot be eliminated, the result must be verified. (2) Conditional pathogenic bacteria can be reported if the coverage of the amplified gene is 100% and if the sequence number is greater than 10. (3) Conditional pathogenic bacteria can be reported if the coverage of the amplified gene is 50%–100% and if the sequence number is greater than 100. The report of pathogenic microorganisms is differed in microorganism species and pathogenic characteristics based on comprehensive clinical judgment.

### 2.5 Pathology

Casts were immersed in formalin solution immediately after removal and sent to the pathology department for hematoxylin and eosin staining and microscopic observation.

### 2.6 Statistical analysis

Quantitative data were represented as mean ± standard (x̅±SD) deviation, and count data were presented as the number of positives. Data between the two groups were analyzed by *t*‐test, chi‐square test and Mann–Whitney *U*‐test, according to the conditions of their statistical tests. *p* < .05 was considered as a significant difference between the two groups. SPSS 22.0 was used for statistical analysis.

## 3 RESULTS

### 3.1 Demographic information

Between January 2023 and August 2023, a total of 35 cases of plastic bronchitis were included in this study. Among these, 15 cases were in the HBoV1‐PB group, and 20 cases were in the MP‐PB group, with ages ranging from 11 months to 6 years. To evaluate the differences in clinical indicators related to the formation of casts following MP infection, we randomly selected 20 age‐matched cases of MP‐PB from patients who underwent bronchoscopy during the same period and were confirmed to have no cast formation.

Furthermore, a total of 52 PB cases related to the two pathogens were diagnosed in the Respiratory Department, as presented in Table 1. This included two cases of PB due to HBoV1 and 15 cases of PB due to MP, in addition to those already accounted for. These cases were excluded from the study as they did not meet the established inclusion and exclusion criteria. The data in the table indicate that a higher number of patients with MP infection underwent bronchoscopy and were diagnosed with PB.

**Table 1:** Proportions of bronchoscopy and PB in infected patients.

Pathogens	PB (%)	*p*	Bronchoscopy (%)	*p*	Total
HBoV1	15 (5.56%)	<.01	76 (28.15%)	<.01	270
MP	35 (6.84%)		164 (32.03%)		512

Abbreviations: HBoV1, human bocavirus type 1; MP, *Mycoplasma pneumoniae*; PB, plastic bronchitis.

**Figure 1:**
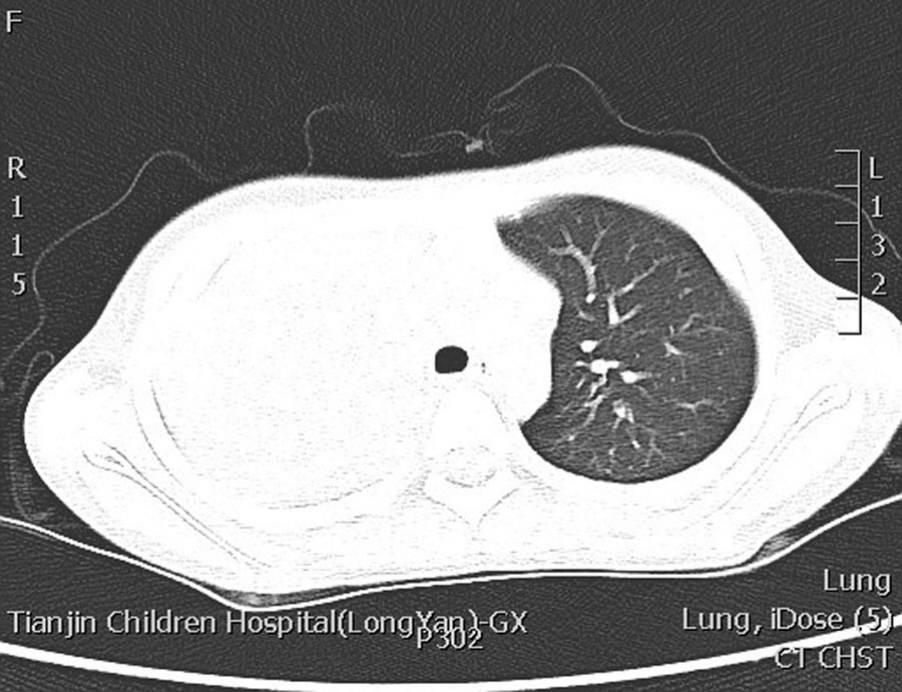
Chest computed tomography of patients showed large consolidated, accompanied with pulmonary atelectasis.

**Figure 2:**
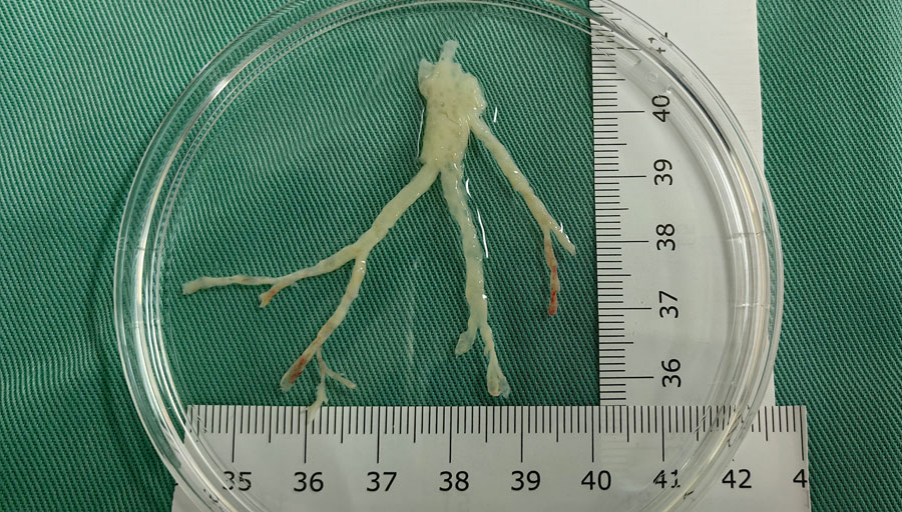
Casts removed by bronchoscopic surgery.

**Figure 3:**
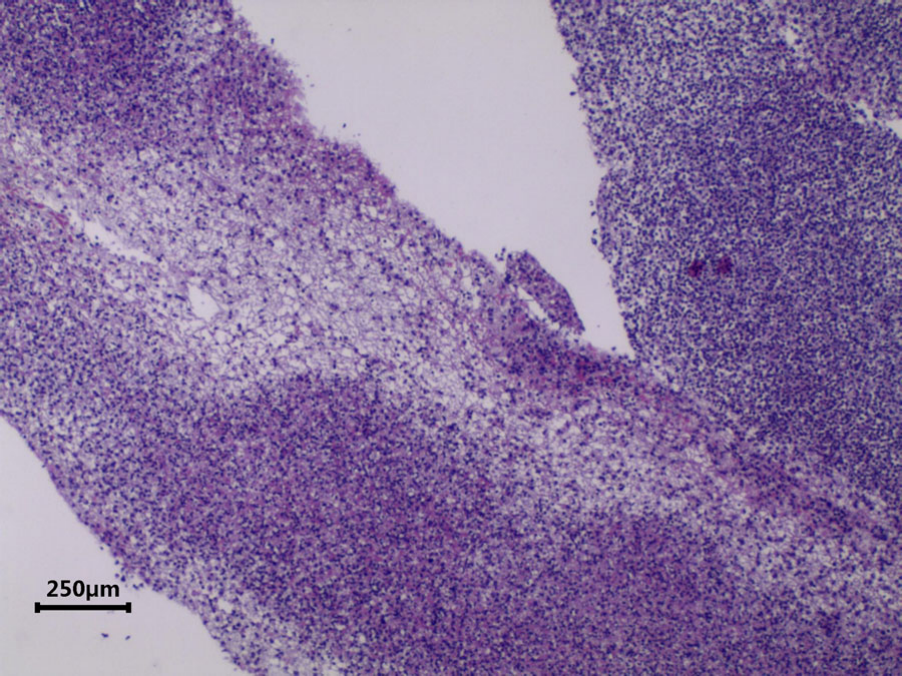
Optical microscopic image of a plastic bronchial cast at 40X magnification. It reveals bifurcated structures, fibrils, and a significant presence of inflammatory cells, indicative of the pathological characteristics of the cast.

**Figure 4:**
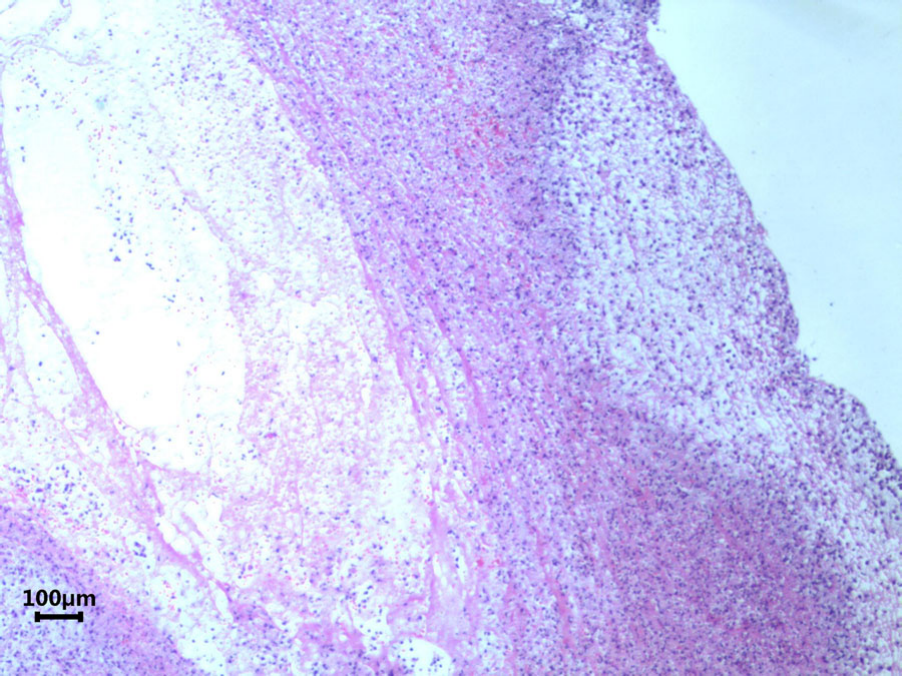
Higher magnification optical microscopic image of a plastic bronchial cast at 100X. The fibrinoid structure is clearly visible, providing a detailed view of the cast's composition.

**Figure 5:**
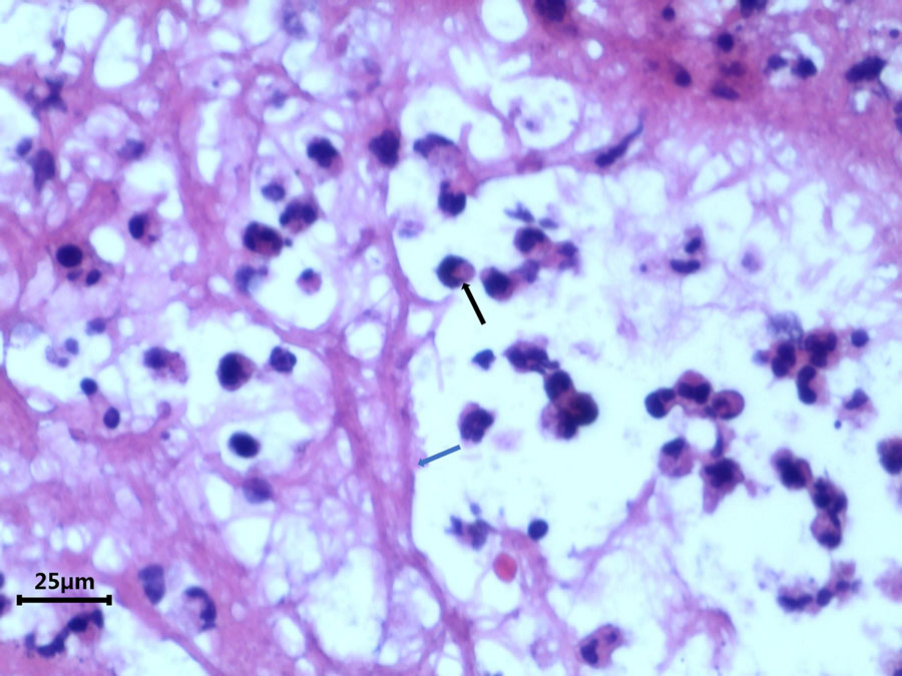
Ultrahigh magnification optical microscopic image of a plastic bronchial cast at 400X. This view highlights the infiltrating inflammatory cells and the surrounding fibrin‐like structures. The black arrow indicates neutrophils within the plastic, while the blue staining represents fibroblasts.

The average age of the HBoV1‐PB group was 2.52 ± 1.65 years, which was significantly younger than that of the MP‐PB group (4.90 ± 1.64 years), *p* < .01. Additionally, the MP‐NPB group, with an average age of 5.10 ± 1.34 years, did not exhibit a statistically significant difference when compared with the MP‐PB group (*p* = .640). Furthermore, while there was no significant difference in body mass index (BMI) between the three groups (*p* = .953). Figures 1, 2, 3, 4, 5 illustrate the chest CT images, casts, and their pathological features.

### 3.2 Clinical features

All patients experienced varying degrees of fever and cough. Wheezing was observed in two patients (13.3%) in the HBoV1‐PB group and none in the MP‐PB group and the MP‐NPB group, with no statistically significant difference. The duration of fever in the HBoV1‐PB group was 3.67 ± 1.11 days, significantly shorter than that in the MP‐PB group (8.73 ± 2.55 days, *p* < .01). Additionally, the maximum temperature recorded in children from the MP‐PB group was significantly higher than that in the HBoV1‐PB group (39.90 ± 0.56°C vs. 39.27 ± 0.66°C, *p* = .04). Detailed information is provided in Table 2 and Table 3.

**Table 2:** Clinical features of HBoV1‐PB and MP‐PB.

	HBoV1‐PB *n* = 15	MP‐PB *n* = 20	*p*	
Demographic information				
Age (years)	**2.52** ± **1.65**	**4.90** ± **1.64**	**<.01**	
BMI (kg/m^2^)	15.78 ± 1.29	15.74 ± 2.12	.953	
Clinical features				
Days of fever (days)	**3.67** ± **1.11**	**8.73** ± **2.55**	**<.01**	
Highest temperature (°C)	**39.27** ± **0.66**	**39.90** ± **0.56**	**.04**	
Wheezing	2 (13.3%)	0	.176	
Image characteristic			
Atelectasis	12 (80.0%)	10 (50.0%)	.069	
Pleural effusion	4 (26.7%)	10 (50.0%)	.163	
Pleural thickening	9 (60.0%)	12 (60.0%)	1.000	
Laboratory examination*				
WBC (/L)	**10.29** ± **2.95**	**8.47** ± **2.95**	**.028**	
Neutrophil%	62.15 ± 17.63	69.10 ± 9.66	.181	
Lymphocyte%	28.75 ± 16.34	22.64 ± 7.58	.194	
Monocyte%	6.48 ± 3.52	6.65 ± 3.28	.886	
CRP (mg/L)	28.58 ± 31.13	48.17 ± 40.48	.126	
LDH (U/L)	382.40 ± 138.46	482.43 ± 174.19	.074	
ALP (U/L)	**238.67** ± **76.64**	**168.13** ± **118.69**	**<.01**	
Fibrinogen (g/L)	**3.95** ± **0.69**	**5.27** ± **2.66**	**.045**	
d‐dimer (mg/L)	**1.01** ± **1.09**	**2.53** ± **2.66**	**<.01**	
Electronic bronchoscopy and BAL sample cytological classification			
Neutrophil%	**35.44** ± **22.42**	**65.55** ± **21.85**	**<.01**	
Eosinophil%	**17.33** ± **12.60**	**1.21** ± **1.26**	**<.01**	
Lymphocyte%	6.22 ± 2.95	7.66 ± 4.34	.388	
Macrophages%	**39.44** ± **12.07**	**24.44** ± **19.06**	**.043**	
Upper lobe	**13 (86.7%)**	**6 (30.0%)**	**<.01**	
Middle lobe (lingula)	0	2 (10.0%)	.496	
Lower lobe	**3 (20.0%)**	**13 (65.0%)**	.016	
The number of operations	**1.33** ± **0.50**	**1.93** ± **0.88**	.011	

Abbreviations: ALP, alkaline phosphatase; CRP, C‐reactive protein; HBoV1, human bocavirus type 1; LDH, lactate dehydrogenase; MP, *Mycoplasma pneumoniae*; PB, plastic bronchitis; PCT, procalcitonin; WBC, white blood cells.

* Reference values for laboratory tests: WBC 4.30–11.30 mg/L, CRP 0.00–8.00 mg/L, LDH 120–300 U/L, ALP 143–406 U/L, fibrinogen 1.800–4.000 g/L, d‐dimer 0.00–0.55 mg/L, and PCT 0–0.05 ng/mL.

When comparing the clinical characteristics of the MP‐PB group to those of the MP‐NPB group, the MP‐NPB group had a fever duration of 7.00 ± 1.33 days, which was significantly shorter than the MP‐PB group's duration (*p* = .024). Additionally, the MP‐NPB group's maximum body temperature was 39.76 ± 0.97°C, and this did not differ significantly from that of the MP‐PB group (*p* = .195).

### 3.3 Image characteristic

Pulmonary solid lesions were present in all patients. Pulmonary atelectasis was observed in 12 children in the HBoV1‐PB group and 10 in the MP‐PB group, without a statistically significant difference (*p* = .069). Additionally, seven cases of atelectasis were noted in the MP‐NPB group, which did not show a significant difference when compared with the MP‐PB group (*p* = .337).

Regarding pleural thickening, there were four cases in the HBoV1‐PB group and 10 cases in the MP‐PB group, with no significant difference between the two groups. The MP‐NPB group had 15 cases of pleural thickening, which was not significantly different from the MP‐PB group when statistical analysis was performed (*p* = .311).

It is noteworthy that there were no cases of pleural effusion in the MP‐NPB group, which is a significant finding given that pleural effusion was present in the MP‐PB group (*p < *.001). But the HBoV1‐PB group showed no difference in the presence of pleural effusion when compared with the MP‐PB group.

### 3.4 Laboratory examination

In the HBoV1‐PB group, the WBC count was 10.29 ± 2.95/L, significantly higher than the MP‐PB group's 8.47 ± 2.95/L (*p* = .028). The HBoV1‐PB group also showed a higher ALP level (238.67 ± 76.64 U/L) compared with the MP‐PB group (168.13 ± 118.69 U/L, *p* < .01), while the MP‐NPB group's ALP (176.50 ± 50.33 U/L) was not significantly different from the MP‐PB group (*p* = .070).

The MP‐PB group exhibited higher levels of fibrinogen (5.27 ± 2.66 g/L) and d‐dimer (2.53 ± 2.66 mg/L), with the latter showing a significant difference from the MP‐NPB group (0.82 ± 1.08 mg/L, *p* < .001). When comparing the d‐dimer and fibrinogen levels between the MP‐NPB and MP‐PB groups, the MP‐PB group had significantly higher d‐dimer levels, but there was no difference in fibrinogen levels.

Among the inflammatory indicators, the MP‐PB group had notably elevated levels of CRP and interleukin‐6, contrasting with the MP‐NPB group's, where significant differences were observed (*p* = .008 and *p* = .022, respectively). The MP‐NPB group's neutrophil percentage (67.32 ± 6.54%) and lymphocyte percentage (24.62 ± 4.62%) were significantly different from those in the MP‐PB group, with lower neutrophils (*p* = .019) and higher lymphocytes (*p* = .009), respectively.

### 3.5 Flexible bronchoscopy and BALF sample cytological classification

All patients underwent flexible bronchoscopy, during which the bronchoscope could be used to identify plastic plugs at various locations blocking the bronchi. Based on the patient's clinical complaints and the extent of plastic plug clearance, the subsequent course of action was determined in accordance with flexible bronchoscopy guidelines. Patients in the MP‐PB group underwent more surgeries compared with those in the HBoV1‐PB group and MP‐NPB group.

A significant difference was noted in the distribution of plastic plugs between the two groups. Most plastic plugs in the HBoV1‐PB group were predominantly located in the upper lobes (*p* < .01), whereas in the MP‐PB group, they were mainly found in the lower lobes (*p* = .016).

Cytological analysis of BALF revealed notable differences in cell populations. Neutrophils predominated in the BALF of the MP‐PB group, while the HBoV1‐PB group had significantly higher proportions of eosinophils and macrophages compared to the MP‐PB group (*p* < .05)

### 3.6 The location of casts under the imaging and bronchoscopy

In a comparison of solid lung lesions observed on chest CT with the location of casts identified during flexible bronchoscopy in 35 cases, we found complete concordance in 30 cases (86%), and partial concordance in five cases (14%), where the CT‐detected area was larger than the area identified via bronchoscopy.

**Table 3:** Clinical features of MP‐PB and MP‐NPB.

	MP‐NPB *n* = 20	MP‐PB *n* = 20	*p*
Demographic information			
Age (years)	5.10 ± 1.34	4.90 ± 1.64	.640
BMI (kg/m^2^)	18.41 ± 2.73	15.74 ± 2.12	.087
Clinical features			
Days of fever (days)	**7.00** ± **1.33**	**8.73** ± **2.55**	**.024**
Highest temperature (°C)	39.76 ± 0.97	39.90 ± 0.56	.195
Image characteristic			
Atelectasis	7 (35%)	10 (50.0%)	.337
Pleural effusion	**0**	**10 (50.0%)**	**<.001**
Pleural thickening	15 (75%)	12 (60.0%)	.311
Laboratory examination			
WBC (/L)	7.60 ± 2.42	8.47 ± 2.95	.431
Neutrophil%	**67.32** ± **6.54**	**69.10** ± **9.66**	**.019**
Lymphocyte%	**24.62** ± **4.62**	**22.64** ± **7.58**	**.009**
CRP (mg/L)	**25.03** ± **18.10**	**48.17** ± **40.48**	**.008**
PCT (ng/mL)	**0.12** ± **0.06**	**0.31** ± **0.46**	**.002**
LDH (U/L)	**274.20** ± **34.53**	**482.43** ± **174.19**	**<.001**
IL‐6 (pg/mL)	**44.30** ± **22.26**	**148.67** ± **127.23**	**.022**
ALP (U/L)	176.50 ± 50.33	168.13 ± 118.69	.070
Fibrinogen (g/L)	4.44 ± 0.65	5.27 ± 2.66	.164
d‐dimer (mg/L)	**0.82** ± **1.08**	**2.53** ± **2.66**	**<.001**
BAL sample cytological classification			
Neutrophil%	76.00 ± 10.66	65.55 ± 21.85	.207
Eosinophil%	0.76 ± 1.55	1.21 ± 1.26	.205
Lymphocyte%	4.90 ± 3.00	7.66 ± 4.34	.064
Macrophages%	17.00 ± 7.82	24.44 ± 19.06	.309
The number of operations	**1.10** ± **0.32**	**1.93** ± **0.88**	**.001**

Abbreviations: ALP, alkaline phosphatase; CRP, C‐reactive protein; IL‐6, interleukin‐6; LDH, lactate dehydrogenase; MP, *Mycoplasma pneumoniae*; PB, plastic bronchitis; PCT, procalcitonin; WBC, white blood cells.

## 4 DISCUSSION

In this study, we further analyzed the proportion of children with MP and HBoV1 who underwent bronchoscopy and had plastic bronchitis confirmed by surgery. Preliminary data indicated a significantly higher proportion of both conditions in children with MP. We then conducted a comparative analysis of PB in children caused by these pathogens. The findings indicated that children in the HBoV1‐PB group were younger and exhibited milder clinical symptoms. In contrast, MP‐PB was associated with elevated inflammatory and coagulation markers, as well as more frequent bronchoscopies, suggesting a more severe disease profile. These results provide novel insights into the distinct pathophysiological characteristics of PB in children, potentially informing clinical treatment approaches.

HBoV1 is a prevalent virus in pediatric respiratory infections, eliciting a corresponding inflammatory response by disrupting epithelial barrier function.^13^ Clinical studies by Zhang et al.^14^ found that the median age of HBoV‐positive children to be 8 months, with a decreasing proportion of positives with increasing age. Tang et al.^15^ reported that HBoV infections were more common in children aged 1–3 years. These findings are consistent with our research results. Notably, children in the HBoV1‐PB group exhibited elevated ALP levels. In an analysis of pediatric bocavirus cases, the authors also reported elevated AST levels in positive patients, indicating liver function involvement.^16^ Although these changes remain the normal range, the potential for HBoV to impact organs such as the liver, kidneys, and heart cannot be excluded. Future follow‐up will monitor organ function indicators to determine the potential for chronic liver damage due to HBoV infection.

Imaging showed a lower proportion of pleural effusion in patients infected with HBoV1, although this difference was not statistically significant, possibly due to our sample size. Considering that children in the MP‐PB group showed higher abnormal coagulation function, while those in the HBoV1‐PB group had significantly fewer fever days and lower maximum body temperature, we speculate that these differences may be due to a weaker inflammatory response in the lungs caused by bocavirus compared to MP.

Interestingly, we found marked differences in cytological classification between the two groups. We attempted to explain why neutrophils predominated in MP‐PB patients, whereas eosinophils were higher in HBoV1‐PB. Yoshida et al.^17^ demonstrated that in influenza virus‐associated PB, eosinophils activate and release extracellular trap networks to clear pathogens. Zhang et al.^18^ reported that when MP and A549 cells were cocultured, the proliferation rate of MP was higher in the group with a high proportion of neutrophils, although neutrophil function was significantly reduced. Whether a similar process, such as neutrophils releasing extracellular traps, can be observed in MP‐PB is a subject for future research.

Further evidence for the presence of neutrophil extracellular traps (NETs) in MP‐PB includes significantly higher fibrinogen and d‐dimer levels in the MP‐PB group compared to the HBoV1‐PB group, with these patients also undergoing more surgeries. Reports of successful PB treatment with tissue plasminogen activator (t‐PA) suggest a link between PB and hypercoagulability.^19^, ^20^, ^21^ In previous studies, *M. pneumoniae* infection was considered to be at risk for thrombosis and embolism, and the level of d‐dimer in children with *M. pneumoniae* pneumonia (MPP) was positively correlated with the severity of the disease.^22^, ^23^ Some studies on NETs have shown that they can act as scaffolds for thrombus formation during coagulation.^24^ Therefore, we hypothesized that the combination of MP and PB may lead to increased hypercoagulation and higher adhesion of casts, necessitating multiple procedures for cast removal.

The current study contributes to the existing body of literature by identifying distinct clinical and laboratory characteristics of plastic bronchitis in preschool children caused by HBoV1 and MP. A notable finding is the marked predominance of neutrophils in both blood and BALF of MP‐infected children, which suggests a heightened inflammatory response and potential involvement of neutrophils in the pathogenesis of MP‐PB. This observation highlights the need for future research to explore the underlying mechanisms of neutrophil activity in MP‐PB, potentially leading to novel therapeutic targets and a deeper understanding of the disease's immunopathogenesis.

There are several limitations in our study. First, this study was a short‐term, single‐center study, and the number of cases was still insufficient. Second, some variables were not evaluated. Third, differences in patient selection and surgical technique among doctors could introduce bias. Therefore, a prospective study with a larger sample size is necessary in the future. Nonetheless, our findings could aid in the recognition and diagnosis of PB in patients due to Human Bocavirus and MP.

## 5 CONCLUSION

In summary, plastic bronchitis is characterized by fever, cough, and possibly wheezing in some patients, with pulmonary CT showing solid lung lesions with or without varying degrees of pulmonary atelectasis. Infections by HBoV1 and MP, the primary causes of plastic bronchitis, result in different clinical manifestations and varying locations of casts.

## AUTHOR CONTRIBUTIONS

**Mengqi Liu**: Conceptualization; data curation; investigation; resources; writing—original draft. **Diwei Wei**: Conceptualization; investigation; methodology; writing—original draft. **Tongqiang Zhang**: Methodology; writing—review and editing. **Yongsheng Xu**: Writing—review and editing. **Wei Guo**: Conceptualization; writing—review and editing.

## CONFLICT OF INTEREST STATEMENT

The authors declare no conflict of interest.

## ETHICS STATEMENT

The protocol for the clinical study was approved by the ethics committees of the Tianjin Children's Hospital (2023‐IITKY‐006). The study was conducted in accordance with the Declaration of Helsinki. The informed consent was obtained from all subjects. Written informed consent was obtained from the parents. The authors affirm that human research participants provided informed consent for publication of the images in figures.

## Supporting information

Supporting information.

## Data Availability

None declared.
